# Discordance in the diagnosis of diabetes: Comparison between HbA1c and fasting plasma glucose

**DOI:** 10.1371/journal.pone.0182192

**Published:** 2017-08-17

**Authors:** Lan T. Ho-Pham, Uyen D. T. Nguyen, Truong X. Tran, Tuan V. Nguyen

**Affiliations:** 1 Bone and Muscle Research Group & Faculty of Applied Sciences, Ton Duc Thang University, Ho Chi Minh City, Vietnam; 2 Department of Internal Medicine, Pham Ngoc Thach University of Medicine, Ho Chi Minh City, Vietnam; 3 Garvan Institute of Medical Research, Sydney, Australia; 4 School of Public Health and Community Medicine, UNSW Australia, Sydney, Australia; 5 University of Technology Sydney (UTS), Sydney, New South Wales, Australia; University of Colorado Denver School of Medicine, UNITED STATES

## Abstract

**Objective:**

HbA1c has been introduced as a complementary diagnostic test for diabetes, but its impact on disease prevalence is unknown. This study evaluated the concordance between HbA1c and fasting plasma glucose (FPG) in the diagnosis of diabetes in the general population.

**Materials and methods:**

The study was designed as a population based investigation, with participants being sampled from the Ho Chi Minh City, Vietnam. Blood samples were collected after overnight fasting and analyzed within 4 hours after collection. HbA1c was measured with high pressure liquid chromatography (Arkray Adams, Japan). FPG was measured by the hexokinase method (Advia Autoanalyzer; Bayer Diagnostics, Germany). Diabetes was defined as HbA1c ≥ 6.5% or FPG ≥ 7.0 mmol/L. Prediabetes was classified as HbA1c between 5.7% and 6.4%.

**Results:**

The study included 3523 individuals (2356 women) aged 30 years and above. Based on the HbA1c test, the prevalence of diabetes and prediabetes was 9.7% (95%CI, 8.7–10.7%; n = 342) and 34.6% (33.0–36.2; n = 1219), respectively. Based on the FPG test, the prevalence of diabetes and prediabetes was 6.3% (95%CI, 5.5–7.2%; n = 223) and 12.1% (11.1–13.2; n = 427). Among the 427 individuals identified by FPG as "pre-diabetes", 28.6% were classified as diabetes by HbA1c test. The weighted kappa statistic of concordance between HbA1c and FPG was 0.55, with most of the discordance being in the prediabetes group.

**Conclusion:**

These data indicate that there is a significant discordance in the diagnosis of diabetes between FPG and HbA1c measurements, and the discordance could have significant impact on clinical practice. FPG appears to underestimate the burden of undiagnosed diabetes.

## Introduction

Type II diabetes and its associated morbidity and mortality are increasingly becoming a serious burden for society in developed as well as developing countries. It is projected that between 2010 and 2030, the number of adults with diabetes will be increased by approximately 20% in developed countries and 69% in developing countries [1]. The Asia Pacific region has been identified as an epicenter of diabetes [2, 3], as the region has undergone rapid economic development accompanied by sedentary lifestyles and poor nutrition: all contributing to the increased risk of the disease. In China alone, the prevalence of diabetes has increased from 1% in 1980 to 10% in 2008 [4, 5]. In Vietnam, epidemiologic studies in Ho Chi Minh City have also recorded a significant increase from 3.8% in 2004 [6] to 12% in 2010 [7], with a majority of diabetes being undiagnosed clinically. Due to late diagnosis and inadequate follow-up or compliance, it is expected that the burden of diabetes in this region will become much greater in the future [8]. Early identification of high-risk individuals is considered the best strategy to contain the burden and costs of diabetes and its associated complications in the general population [9].

Previously, fasting plasma glucose (FPG) was used as a criterion for diagnosing diabetes, alone or during an oral glucose tolerance test where 2-h level is also a criterion. This method is sensitive, but has poor reproducibility and patients have to fast overnight prior to either test. In recent years, glycated hemoglobin (HbA1c), initially discovered by Rahbar et al in 1969 [10], was recommended as a tool for the diagnosis and monitoring of treatment of diabetes. In 2009, the International Expert Committee recommended using an HbA1c ≥ 6.5% (48 mmol/mol) to diagnose diabetes [11], and this position was also endorsed by the American Diabetes Association [12]. There is evidence that mean HbA1c levels are different between ethnicities, with Asians having higher levels than Whites [13], and this difference could result in ethnic-related difference in the estimation of prevalence of diabetes. However, there have been few studies in Asia Pacific to evaluate the prevalence of diabetes using the proposed criterion of HbA1c.

In this study, we sought to evaluate the concordance between the new HbA1c and old FPG criteria in the diagnosis of diabetes. In addition, we investigated the risk factors for undiagnosed diabetes in men and women.

## Study design and methods

### Study design

This study was part of the Vietnam Osteoporosis Study (VOS) which was initiated in mid-2015. The protocol and design of VOS have been published elsewhere [14]. Briefly, VOS is designed as a population based, long-term prospective study, with the setting being Ho Chi Minh City (formerly Saigon). The City is a major economic hub of the ASEAN region, with a population of 8.2 million. The study's procedure and protocol were approved by the research and ethics committee of the People's Hospital 115. The study was conducted according to the ethical principles of the Declaration of Helsinki, and all participants gave written informed consent.

The inclusion criteria were men and women aged between 20 and 90 years, who agreed to participate in the Study. We excluded individuals who were deemed to have impaired cognitive function or were not willing to give informed consent or were physically unable to complete clinical tests.

We used two approaches to recruit participants. In the first approach, we contacted community organizations to solicit a list of members, and from the list we ran a computer program to randomly select individuals who met the age and gender criteria. A letter was then sent to the selected individuals to invite them and their family members to participate in the Study. In the second approach, we recruited participants via television, the Internet, and flyers in universities. The flyers described (in Vietnamese) the study's purposes, procedures, and benefits of participants. Individuals who agreed to participate in the study were then transported to the Bone and Muscle Research Laboratory at the Ton Duc Thang University for clinical assessment and evaluation. The participants did not receive any financial incentive, but received a free health check-up, and lipid analyses.

### Data collection and measurements

Each participant was administered a structured questionnaire by a trained interviewer. The questionnaire solicited information concerning clinical history, medication use, lifestyle factors, history of falls and fractures, and anthropometric factors. Height and weight were measured by an electronic portable, wall-mounted stadiometer (Seca Model 769; Seca Corp, CA, USA) without shoes, ornaments, hats or heavy layers of clothing. Body mass index (BMI) was derived as the weight in kilograms divided by the square of the height in meters.

Waist circumference (WC) and hip circumference (HC) were also measured in each participant by using the WHO protocol [15]. HC was measured around the widest portion of the buttocks (in standing position) by using a measuring tape. WC was measured at the midpoint between lower margin of the least palpable rib and the top of the iliac crest. Waist to hip ratio (WHR) was derived as the ratio of WC over HC. Central obesity was defined as WHR > 0.85 for women or >0.90 for men.

Participants were also asked to provide information on current and past smoking habits. Alcohol intake in average numbers of standard drinks per day, at present as well as within the last 5 years, was obtained. Clinical data including blood pressure, pulse, and reproductive history (i.e. parity, age of menarche and age of menopause), medical history (i.e. previous fracture, previous and current use of pharmacological therapies) were also obtained. Two blood pressure measurements were taken (5 min apart) in seated position, and the mean of two measurements was taken as the individual's blood pressure. Individuals were classified as having hypertension if their average systolic blood pressure ≥ 140 mmHg or diastolic blood pressure ≥ 90 mmHg.

Whole body bone density was measured using a Hologic Horizon (Hologic Corp, Bedford, MA, USA). The densitometer was standardized by phantom before each measurement. The measurement was done by a qualified radiology technologist. Based on 20 individuals, the coefficient of variation in BMD at our lab was 1.5%. Fat mass and lean mass were derived from the whole body scan.

### Laboratory analyses

A venous blood sample (12 ml) was taken from each participant by venepuncture between 7:00 AM and 11:00 AM. The serum was immediately frozen to -20°C prior to biochemical analysis which was done within 24 hours after the collection. HbA1c levels were measured with high pressure liquid chromatography (HPLC) analyzers ADAMS^™^ A_1c_ HA-8160 (Arkray, Kyoto, Japan). The intra- and inter-assay coefficient of variation for this HPLC is less than 1%. FPG levels were determined by the hexokinase method (Advia 1800 Autoanalyzer; Bayer Diagnostics, Leverkusen, Germany) with an intra-measurement coefficient of variance of 0.98–1.34%. Total cholesterol, LDL cholesterol and triglycerides were measured by an enzymatic calorimetric test (Advia 1800, Autoanalyzer; Bayer Diagnostics). The intraassay coefficient of variation was 0.6% for cholesterol and 1.6% for triglycerides. All specimens were analyzed at the MEDIC's Department of Biochemistry and Paraclinical Services (Ho Chi Minh City, Vietnam).

An individual was diagnosed to have diabetes if the individual's HbA1c value ≥ 6.5% (48 mmol/mol) [11]. Pre-diabetes was defined as HbA1c value between 5.7% and 6.4% (39–46 mmol/mol) [12]. In addition and for comparison, diabetes was also diagnosed as individuals with FPG ≥ 7.0 mmol/L, and pre-diabetes was classified as FPG between 5.6 and 6.9 mmol/L (i.e., impaired fasting glucose).

### Data analysis

Point prevalence of diabetes and pre-diabetes was estimated for each 10-year age group and gender. Ninety-five confidence interval of a proportion was determined by a Bayesian method using the "binom" package within the R program. The concordance in the diagnoses of diabetes between HbA1c and FPG was assessed by the Kappa statistic. In the next analysis, we used the logistic regression method to model the relationship between the risk of diabetes (outcome) and potential risk factors, including age, blood pressure, and measures of obesity. The strength of association was expressed in odds ratio (OR) and 95% confidence interval. The discrimination of diabetes vs non-diabetes was assessed by the c-statistic [16]. All statistical analyses were conducted using the R statistical environment [17].

## Results

### Characteristics of participants

The study has enrolled 2356 women and 1167 men aged 30 years and older (at study entry), whose baseline characteristics stratified by gender are shown in Table 1. The average age of participants was 52 years, with no significant difference between women and men. About 46% of men and 3% of women self-reported that they were current smokers. A majority of participants (~77% of women and 72% of men) had secondary or primary education.

**Table 1:** Characteristics of 2356 women and 1167 men.

Variable	Women	Men	P-value
Number of subjects	2356	1167	
Marital status			<0.0001
• Married (%)	1816 (77.2)	1073 (91.9)	
• Single (%)	287 (12.2)	66 (5.7)	
• Divorced (%)	80 (3.4)	14 (1.2)	
• Widowed (%)	170 (7.2)	14 (1.2)	
Educational attainment			<0.0001
• Primary (%)	589 (25.0)	182 (15.6)	
• Secondary (%)	1216 (51.6)	658 (56.4)	
• College and university (%)	551 (23.4)	327 (28.0)	
Current smoking (Yes)	25 (1.1)	541 (46.4)	<0.0001
Current use of alcohol	68 (2.9)	569 (48.8)	<0.0001
Age (years)	52.4 (11.4)	51.1 (11.9)	0.184
Height (cm)	152.5 (5.3)	163.3 (5.9)	<0.0001
Weight (kg)	53.7 (8.2)	62.6 (9.9)	<0.0001
Body mass index (kg/m^2^)	23.1 (3.2)	23.4 (3.2)	0.002
Percent body fat (%)	41.9 (5.2)	30.6 (5.4)	<0.0001
Fat mass (kg)	23.1 (5.6)	19.5 (5.7)	<0.0001
Lean mass (kg)	31.5 (4.4)	43.2 (6.2)	<0.0001
Waist to hip ratio	0.87 (0.07)	0.91 (0.07)	<0.0001
HbA1c (%)	5.77 (0.9)	5.76 (0.87)	0.631
Glucose (mmol/L)	5.31 (1.58)	5.35 (1.39)	0.412

The mean BMI of the participants was 23.2 (SD 3.2) kg/m^2^. Overall, 199 women (8.4%) and 123 men (11%) had BMI ≥ 27.5 kg/m^2^, the level considered "obese" by Asian criteria. However, 60% of women and men were classified as having abdominal obesity. As expected, women had greater fat mass but lower lean mass than men. However, there was no statistically significant difference in HbA1c and FPG between women and men.

Using the cut-off value of HbA1c ≥ 6.5% (48 mmol/mol), the prevalence of diabetes in women and men was 9.9% (95% CI, 8.8–11.2; n = 234) and 9.3% (95% CI, 7.7 to 11.1; n = 108), respectively. There was no significant difference in the prevalence between women and men (*P* = 0.79). Among the 342 individuals with diabetes, only 130 (38%) were clinically known to have the disease and were on diabetic treatment. However, using the criterion of FPG ≥ 7 mmol/L, the prevalence of diabetes was 6.2% (95% CI, 5.2 to 7.2) in women and 6.6% (95% CI, 5.3 to 8.3) in men. If both HbA1c and FPG criteria were used, the prevalence of diabetes was increased to 10.4% (95% CI, 9.2 to 11.7) in women and 10.1% (95% CI, 8.5 to 12.0) in men (Table 2).

**Table 2:** Prevalence of diabetes based on HbA1c and fasting plasma glucose (FPG) stratified by gender.

Classification	Women		Men	
	No. of subjects	Prevalence (%)	No. of subjects	Prevalence (%)
**Based on HbA1c**				
• Normal	1306	55.4	656	56.2
• Pre-diabetes	816	34.7	403	34.5
• Diabetes	234	9.9	108	9.3
**Based on FPG**				
• Normal	1931	81.9	942	80.7
• Pre-diabetes	279	11.9	148	12.7
• Diabetes	146	6.2	77	6.6
**Based on HbA1c or FPG**				
• Non-diabetes	2108	89.5	1049	89.9
• Diabetes	248	10.5	118	10.1

### Concordance between HbA1c and FPG

**Fig 1:**
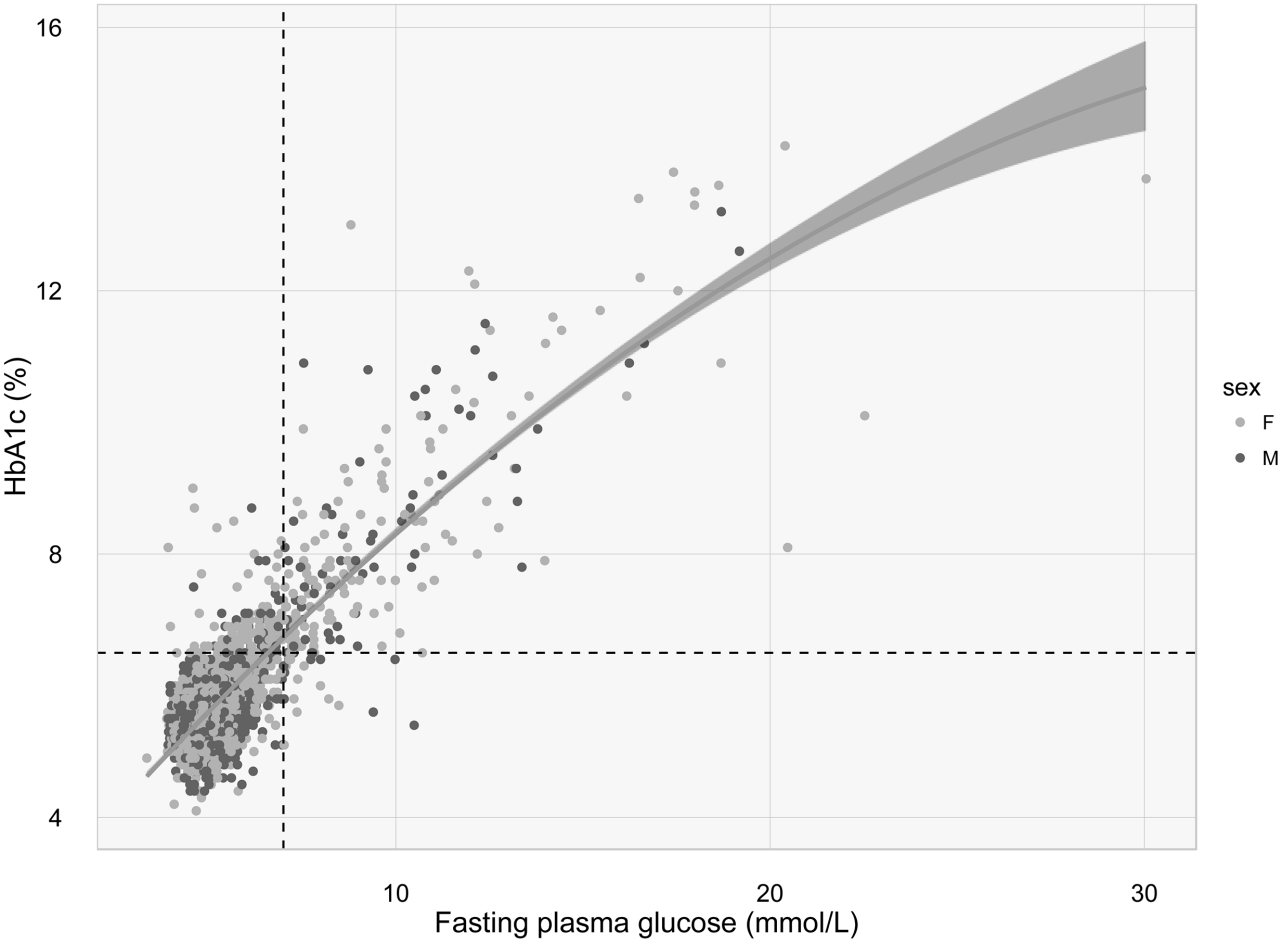
Relationship between fasting plasma glucose and HbA1c. The dotted lines represent the threshold for the diagnosis of diabetes by FPG (7 mmol/L) and by HbA1c (6.5%).

There was a correlation between HbA1c and FPG (*r* = 0.84; P < 0.0001), but the relationship between the two measurements was more consistent with a quadratic function (Fig 1).

Because there was no perfect correlation between the two measurements, discordance in the diagnoses is expected. Indeed, among 342 individuals classified by HbA1c as having diabetes, 202 (59.1%) were classified as having the same diagnosis by FPG. Among 1219 individuals classified as having pre-diabetes by HbA1c, FPG test provided a similar diagnosis for only ~20% (Table 3).

**Table 3:** Concordance in diagnostic classifications between HbA1c and FPG.

Diagnosis based on FPG	Diagnosis based on HbA1c		
	Normal	Pre-diabetes	Diabetes
Normal	**1863 (95.2)**	963 (79.0)	42 (12.33)
Pre-diabetes	90 (4.6)	**239 (19.6)**	98 (28.6)
Diabetes	4 (0.2)	17 (1.4)	**202 (59.1)**

Notes: Numbers in brackets are percentages of columwise total. This analysis was based on data from 3523 individuals whose data were available for both HbA1c and FPG.

Diabetes diagnosed by FPG≥7 mmol/L had a sensitivity of 60% and a specificity of 89% compared with HbA1c 6.5% (48 mmol/mol) or more for diagnosing diabetes. Overall, the weighed kappa statistic was 0.55 (95% CI, 0.52 to 0.58).

### Risk factors for diabetes

In logistic regression analysis, the risk of diabetes was significantly associated with gender (i.e., men had lower risk than women), advancing age, greater BMI and greater WHR (Table 4).

**Table 4:** Risk factors for diabetes (based on HbA1c): Results of logistic regression analysis.

Risk factor	Unit of comparison	Odds ratio (95% confidence interval)	P-value
**Model I**			
Gender	Men / women	0.75 (0.57–0.98)	0.036
Age	+5 years	1.30 (1.23–1.37)	<0.001
BMI	+ 1 kg/m^2^	1.15 (1.11–1.19)	<0.001
WHR	+ 0.1	1.81 (1.51–2.17)	<0.001
**Model II**			
FPG	1 mmol/L	9.34 (7.46–11.70)	<0.001
Age	+5 years	1.27 (1.17–1.38)	<0.001
WHR	+ 0.1	1.54 (1.19–2.00)	<0.001

**Fig 2:**
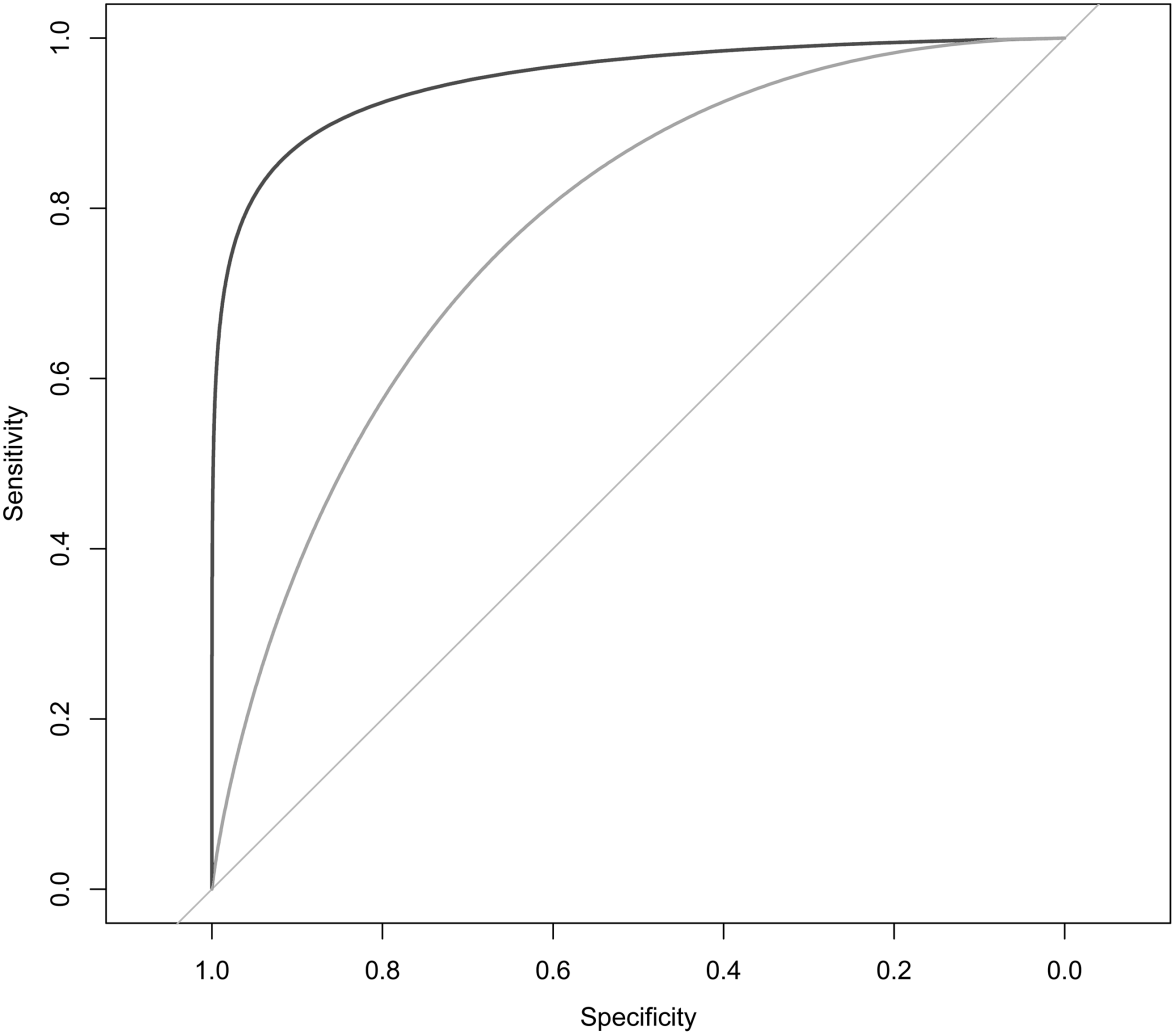
Area under the receiver operating characteristic curve for model I (gender, age, body mass index and waist-to-hip ratio; blue line), and model II (age, waist-to-hip ratio, and glucose; red line). The area under the ROC curve for model I was 0.776, and model II 0.956.

It is interesting to observe that greater BMI (OR per 1 kg/m^2^ 1.15; 95%CI, 1.11 to 1.19) and greater WHR (OR per 0.1 unit: 1.81; 95%CI, 1.51 to 2.17) were independent predictors of diabetes risk. The area under the ROC curve of this model was 0.77 (Fig 2).

An alternative model for predicting the risk of diabetes included FPG, age and WHR. In this model, each mmol/L increase in FPG was associated with 9.3-fold increase in the odds of diabetes (OR 9.3; 95% CI, 7.4 to 11.7). The AUC of this model was 0.96 (Fig 2).

## Discussion

Since the introduction of HbA1c as a diagnostic test for diabetes into clinical practice, its impact on the estimate of prevalence of diabetes has not been systematically documented in Asian populations. In this study, based on the HbA1c criteria, we found that the prevalence of diabetes (~10%) and pre-diabetes (~35%) has reached an epidemic level in an urban Vietnamese population, but there was a significant discordance in the classification of diabetes between HbA1c and FPG criteria. Our findings deserve further elaboration.

Our finding demonstrates a rapid increase in the prevalence of diabetes in Vietnam. In 2004, a population based study using FPG test found that 3.8% of the Ho Chi Minh City (the present study setting) had diabetes [6]. This prevalence was increased to 7% in 2008 [18]. In 2010, in a random sample of 2142 individuals in the same city, it was found that 11% of men and 12% had undiagnosed diabetes using FPG criteria [7], and these estimates are very comparable with our estimates (~10%). Taken together, previous data and our present findings suggest that the prevalence of diabetes in Vietnam has been continuously increasing [19]. This increasing trend was also observed in Japan [20], China [5], Korea [21], and Thailand [22] which is thought to be associated with the pace of urbanization of those countries during the past three decades.

An important finding from this study is the discordance between HbA1c and FPG in the diagnosis of diabetes. The discordance was mainly in the category of diabetes and pre-diabetes, not the normal group. For example, among those with diabetes by HbA1c criteria, only 59% were classified as having the diagnosis by FPG criteria. However, among the normal group by HbA1c criteria, 95% were also normal by FPG criteria. Thus, overall, HbA1c identified more people at risk of diabetes than did FPG. This observation has also been noted in other Asian populations [23–25] and an African population [26], but not in US adults [27]. HbA1c is considered to be an indicator of long-term glycemic exposure and can therefore reflect the average glucose concentration over the preceding 2–3 months [28, 29]. Thus, the use of HbA1c can avoid the problem of within-subject fluctuation in glucose measurements [30]. At the population level, our finding implies that HbA1c may be a more sensitive test to identify at-risk individuals in Asian populations.

It is, however, also possible that HbA1c and FPG identify two different groups of diabetic individuals. In a study on Japanese individuals, FPG, 2-hour plasma glucose and HbA1c were each strongly associated with retinopathy, and all three measures had similar sensitivity, specificity and degree of discrimination [31]. We also observed a strong correlation (r = 0.84) between FPG and HbA1c, and this finding is highly agreeable to previous studies [31, 32]. Nevertheless, at the individual level, the imperfect correlation means that there are substantial differences in the classification of diabetes between HbA1c and FPG measurements. Theoretically, FPG and HbA1c provide different information; the former measures the blood glucose at a point in time, whereas the latter reflects long-term glucose control. It is, therefore, not surprising that the individuals classified as diabetic by FPG may be normal by HbA1c test. Our data are consistent with the view that a value of HbA1c may signify a false negative finding of diabetes [30].

We also note that among those with HbA1c confirmed diabetes, only 30% had come to clinical attention or were on diabetic medications. In the developed world approximately 50% of individuals with diabetes are clinically undiagnosed [33]. This finding underscores a difficult aspect of diabetes management as the disease is often asymptomatic at its onset and can remain undiagnosed for several years while complications are developing [6, 34]. In practice, there have been concerns on the appropriateness of HbA1c as a means for diagnosing diabetes in Asians [35]. However, a large cross-sectional study in three Asian ethnicities in Singapore using retinopathy as an outcome conclude that the HbA1c threshold of 6.5% is appropriate for the diagnosis of diabetes in Asians [36].

In this study, we found that apart from age and body mass index, greater waist to hip ratio was an independent risk factor for diabetes, and this is consistent with our previous study [7]. We proposed two possible models for identifying individuals at high-risk of having diabetes. One model uses gender, age, body mass index, and waist to hip ratio as predictor variables, but this model has modest discrimination (AUC = 0.78). The other model uses only three factors: age, waist to hip ratio, and fasting plasma glucose, and this model has a very good discrimination (AUC 0.96). Thus, in the absence of HbA1c, it is possible to use fasting plasma glucose together with age and central obesity to accurately predict the risk of diabetes for an individual.

The findings of this study should of course be considered in context of strengths and weaknesses. The study was based on a large, well characterized cohort who was recruited without any specific criteria that would affect the risk of diabetes. Thus, the estimates presented here are likely to reflect the true prevalence in the general population. The HbA1c and FPG tests were done in a high quality laboratory using latest technologies. To our knowledge, this is the first population-based study in Vietnam that used HbA1c to estimate the prevalence of diabetes. However, the study was designed as a cross-sectional investigation, and we could not estimate the incidence of diabetes. Also, no cause-and-effect inference could be drawn from the association between diabetes and risk factors. We did not ascertain retinopathy and neuropathy in the study, and as a result, we could not determine whether HbA1c or FPG is a more accurate diagnostic test of diabetes. The present data were drawn from a highly urban population, therefore the findings may not be generalizable to the rural population.

In summary, in this urban Vietnamese population, the prevalence of undiagnosed diabetes has reached an epidemic proportion of 10%, a 2.5-fold increase from ~4% of 10 years ago. Our data also suggest that there is a substantial disparity in the classification of diabetes between fasting plasma glucose test and Hemoglobin A1c test, and the disparity can have impact on the eventual cost and burden of missed diabetes and its complications.
